# 3-Chloro-*N*′-(2,4-dichloro­benzyl­idene)benzohydrazide

**DOI:** 10.1107/S1600536810051913

**Published:** 2010-12-18

**Authors:** Yan Lei

**Affiliations:** a School of Chemistry & Environmental Engineering, Chongqing Three Gorges University, Chongqing 404000, People’s Republic of China

## Abstract

The title compound, C_14_H_9_Cl_3_N_2_O, adopts an *E* configuration about the methyl­idene unit and the two aromatic rings form a dihedral angle of 6.6 (2)°. In the crystal, mol­ecules are linked *via* N—H⋯O hydrogen bonds, forming *C*(4) chains propagating in [001]. C—H⋯O inter­actions reinforce the chains.

## Related literature

For background to hydrazones, see: El-Asmy *et al.* (2010 ▶); El-Sherif (2009 ▶); Singh *et al.* (2009 ▶); El-Tabl *et al.* (2007 ▶). For structures of hydrazone compounds, see: Qiao *et al.* (2010 ▶); Hussain *et al.* (2010 ▶); Han & Zhao (2010 ▶); Ahmad *et al.* (2010 ▶).
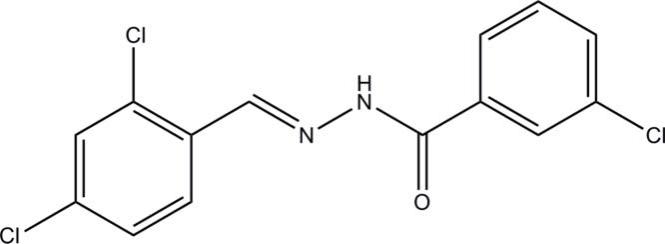


## Experimental

### 

#### Crystal data

C_14_H_9_Cl_3_N_2_O
*M* *_r_* = 327.58
Monoclinic, 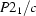

*a* = 12.372 (3) Å
*b* = 14.071 (2) Å
*c* = 8.332 (2) Å
β = 97.582 (3)°
*V* = 1437.9 (5) Å^3^
*Z* = 4
Mo *K*α radiation
μ = 0.63 mm^−1^
*T* = 298 K
0.30 × 0.27 × 0.27 mm

#### Data collection

Bruker SMART CCD diffractometer
Absorption correction: multi-scan (*SADABS*; Bruker, 2000 ▶) *T*_min_ = 0.833, *T*_max_ = 0.848
7125 measured reflections
3081 independent reflections
1776 reflections with *I* > 2σ(*I*)
*R*_int_ = 0.042

#### Refinement

*R*[*F* ^2^ > 2σ(*F* ^2^)] = 0.054
*wR*(*F* ^2^) = 0.129
*S* = 1.02
3081 reflections
184 parameters
1 restraint
H atoms treated by a mixture of independent and constrained refinement
Δρ_max_ = 0.27 e Å^−3^
Δρ_min_ = −0.34 e Å^−3^

### 

Data collection: *SMART* (Bruker, 2000 ▶); cell refinement: *SAINT* (Bruker, 2000 ▶); data reduction: *SAINT*; program(s) used to solve structure: *SHELXS97* (Sheldrick, 2008 ▶); program(s) used to refine structure: *SHELXL97* (Sheldrick, 2008 ▶); molecular graphics: *SHELXTL* (Sheldrick, 2008 ▶); software used to prepare material for publication: *SHELXTL*.

## Supplementary Material

Crystal structure: contains datablocks global, I. DOI: 10.1107/S1600536810051913/hb5766sup1.cif

Structure factors: contains datablocks I. DOI: 10.1107/S1600536810051913/hb5766Isup2.hkl

Additional supplementary materials:  crystallographic information; 3D view; checkCIF report

## Figures and Tables

**Table 1:** Hydrogen-bond geometry (Å, °)

*D*—H⋯*A*	*D*—H	H⋯*A*	*D*⋯*A*	*D*—H⋯*A*
N2—H2⋯O1^i^	0.90 (1)	2.08 (2)	2.900 (3)	152 (3)
C7—H7⋯O1^i^	0.93	2.35	3.155 (3)	145

Symmetry code: (i) 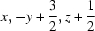.

**Fig. 1.**
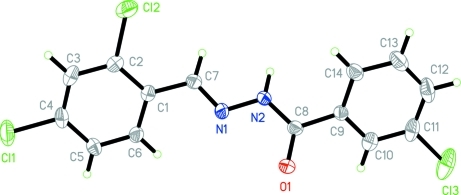
The molecular structure of the title compound with 30% probability displacement ellipsoids.

**Fig. 2.**
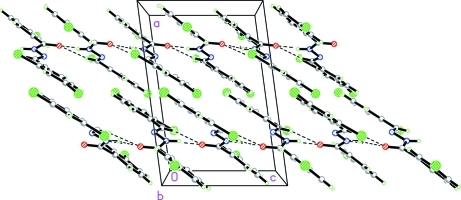
The packing of the title compound. Hydrogen bonding is shown in dashed lines.

## supplementary crystallographic information

### Comment

Significant attention has been attracted on the hydrazones for their biological properties, coordinative capability, and applications in analytical chemistry (El-Asmy *et al.*, 2010; El-Sherif, 2009; Singh *et al.*, 2009; El-Tabl *et al.*, 2007). Recently, a number of hydrazones have been prepared and investigated for their structures (Qiao *et al.*, 2010; Hussain *et al.*, 2010; Han & Zhao, 2010; Ahmad *et al.*, 2010). As a continuation of hydrazones, the author reports herein the title new compound.

The molecule of the title compound, Fig. 1, adopts an *E* configuration about the methylidene unit. The two aromatic rings form a dihedral angle of 6.6 (2)°. In the crystal, the molecules are linked *via* intermolecular N—H···O and C—H···O hydrogen bonds (Table 1), to form chains at the *c*-axis direction (Fig. 2).

### Experimental

3-Chlorobenzohydrazide (1 mmol, 0.170 g) was dissolved in methanol (50 ml), then 2,4-dichlorobenzaldehyde (1 mmol, 0.174 g) was added into the solution. The reaction mixture was heated under reflux for 1 h and cooled to room temperature. Colourless needle-shaped crystals were formed by slow evaporation of the solvent for a week.

### Refinement

The amino H atom was located in a difference Fourier map and refined isotropically, with the N—H distance restrained to 0.90 (1) Å. Other H atoms were positioned geometrically and constrained to ride on their parent atoms, with C—H = 0.93 Å, and with *U*_iso_(H) = 1.2*U*_eq_(C).

### Crystal data

**Table:**

C_14_H_9_Cl_3_N_2_O	*F*(000) = 664
*M**_r_* = 327.58	*D*_x_ = 1.513 Mg m^−^^3^
Monoclinic, *P*2_1_/*c*	Mo *K*α radiation, λ = 0.71073 Å
*a* = 12.372 (3) Å	Cell parameters from 1072 reflections
*b* = 14.071 (2) Å	θ = 2.3–25.1°
*c* = 8.332 (2) Å	µ = 0.63 mm^−^^1^
β = 97.582 (3)°	*T* = 298 K
*V* = 1437.9 (5) Å^3^	Cut from needle, colourless
*Z* = 4	0.30 × 0.27 × 0.27 mm

### Data collection

**Table:**

Bruker SMART CCD diffractometer	3081 independent reflections
Radiation source: fine-focus sealed tube	1776 reflections with *I* > 2σ(*I*)
graphite	*R*_int_ = 0.042
ω scans	θ_max_ = 27.0°, θ_min_ = 2.2°
Absorption correction: multi-scan (*SADABS*; Bruker, 2000)	*h* = −15→11
*T*_min_ = 0.833, *T*_max_ = 0.848	*k* = −17→17
7125 measured reflections	*l* = −10→10

### Refinement

**Table:**

Refinement on *F*^2^	Primary atom site location: structure-invariant direct methods
Least-squares matrix: full	Secondary atom site location: difference Fourier map
*R*[*F*^2^ > 2σ(*F*^2^)] = 0.054	Hydrogen site location: inferred from neighbouring sites
*wR*(*F*^2^) = 0.129	H atoms treated by a mixture of independent and constrained refinement
*S* = 1.02	*w* = 1/[σ^2^(*F*_o_^2^) + (0.0422*P*)^2^ + 0.3462*P*] where *P* = (*F*_o_^2^ + 2*F*_c_^2^)/3
3081 reflections	(Δ/σ)_max_ = 0.001
184 parameters	Δρ_max_ = 0.27 e Å^−^^3^
1 restraint	Δρ_min_ = −0.34 e Å^−^^3^

### Special details

**Table:**

Geometry. All e.s.d.'s (except the e.s.d. in the dihedral angle between two l.s. planes) are estimated using the full covariance matrix. The cell e.s.d.'s are taken into account individually in the estimation of e.s.d.'s in distances, angles and torsion angles; correlations between e.s.d.'s in cell parameters are only used when they are defined by crystal symmetry. An approximate (isotropic) treatment of cell e.s.d.'s is used for estimating e.s.d.'s involving l.s. planes.
Refinement. Refinement of *F*^2^ against ALL reflections. The weighted *R*-factor *wR* and goodness of fit *S* are based on *F*^2^, conventional *R*-factors *R* are based on *F*, with *F* set to zero for negative *F*^2^. The threshold expression of *F*^2^ > σ(*F*^2^) is used only for calculating *R*-factors(gt) *etc*. and is not relevant to the choice of reflections for refinement. *R*-factors based on *F*^2^ are statistically about twice as large as those based on *F*, and *R*- factors based on ALL data will be even larger.

### Fractional atomic coordinates and isotropic or equivalent isotropic displacement parameters (Å^2^)

**Table:**

	*x*	*y*	*z*	*U*_iso_*/*U*_eq_	
Cl1	0.50321 (9)	1.17766 (6)	0.13090 (13)	0.0852 (4)	
Cl2	0.27379 (8)	1.10398 (6)	0.61537 (13)	0.0791 (3)	
Cl3	0.12211 (9)	0.31908 (6)	0.63795 (16)	0.1013 (5)	
N1	0.27659 (18)	0.81292 (16)	0.4682 (3)	0.0403 (6)	
N2	0.22918 (19)	0.74940 (16)	0.5631 (3)	0.0408 (6)	
O1	0.20262 (17)	0.64606 (14)	0.3554 (2)	0.0544 (6)	
C1	0.3473 (2)	0.96532 (19)	0.4329 (3)	0.0384 (7)	
C2	0.3406 (2)	1.0619 (2)	0.4604 (4)	0.0456 (8)	
C3	0.3859 (2)	1.1283 (2)	0.3661 (4)	0.0528 (8)	
H3	0.3788	1.1930	0.3845	0.063*	
C4	0.4413 (2)	1.0962 (2)	0.2456 (4)	0.0503 (8)	
C5	0.4516 (2)	1.0012 (2)	0.2160 (4)	0.0503 (8)	
H5	0.4903	0.9805	0.1342	0.060*	
C6	0.4041 (2)	0.9370 (2)	0.3086 (3)	0.0435 (7)	
H6	0.4102	0.8724	0.2874	0.052*	
C7	0.2964 (2)	0.8950 (2)	0.5281 (4)	0.0418 (7)	
H7	0.2790	0.9101	0.6302	0.050*	
C8	0.1933 (2)	0.66637 (19)	0.4955 (4)	0.0396 (7)	
C9	0.1403 (2)	0.59954 (19)	0.5991 (3)	0.0361 (6)	
C10	0.1539 (2)	0.5032 (2)	0.5755 (4)	0.0475 (8)	
H10	0.1974	0.4820	0.4999	0.057*	
C11	0.1030 (3)	0.4393 (2)	0.6640 (4)	0.0582 (9)	
C12	0.0359 (3)	0.4696 (3)	0.7725 (4)	0.0723 (11)	
H12	0.0004	0.4258	0.8307	0.087*	
C13	0.0219 (3)	0.5651 (3)	0.7939 (4)	0.0663 (10)	
H13	−0.0235	0.5860	0.8671	0.080*	
C14	0.0743 (2)	0.6307 (2)	0.7086 (3)	0.0490 (8)	
H14	0.0650	0.6954	0.7250	0.059*	
H2	0.227 (2)	0.763 (2)	0.6680 (15)	0.059*	

### Atomic displacement parameters (Å^2^)

**Table:**

	*U*^11^	*U*^22^	*U*^33^	*U*^12^	*U*^13^	*U*^23^
Cl1	0.1072 (8)	0.0612 (6)	0.0882 (8)	−0.0214 (5)	0.0174 (6)	0.0292 (5)
Cl2	0.0891 (7)	0.0575 (6)	0.0975 (8)	0.0031 (5)	0.0376 (6)	−0.0209 (5)
Cl3	0.1025 (8)	0.0464 (5)	0.1416 (11)	−0.0168 (5)	−0.0342 (7)	0.0293 (6)
N1	0.0504 (14)	0.0348 (13)	0.0360 (15)	−0.0021 (11)	0.0066 (11)	0.0053 (11)
N2	0.0556 (15)	0.0390 (13)	0.0290 (13)	−0.0088 (11)	0.0094 (12)	−0.0006 (12)
O1	0.0815 (15)	0.0482 (12)	0.0366 (13)	−0.0143 (11)	0.0201 (11)	−0.0062 (10)
C1	0.0382 (15)	0.0357 (16)	0.0393 (17)	−0.0020 (13)	−0.0017 (13)	0.0028 (13)
C2	0.0452 (17)	0.0405 (17)	0.050 (2)	0.0006 (13)	0.0034 (14)	−0.0049 (15)
C3	0.0559 (19)	0.0327 (16)	0.066 (2)	−0.0040 (14)	−0.0073 (17)	0.0029 (16)
C4	0.0522 (18)	0.0457 (18)	0.051 (2)	−0.0096 (15)	−0.0022 (16)	0.0176 (16)
C5	0.0604 (19)	0.049 (2)	0.042 (2)	−0.0016 (15)	0.0087 (15)	0.0100 (15)
C6	0.0555 (18)	0.0354 (15)	0.0388 (18)	−0.0012 (13)	0.0025 (14)	0.0029 (14)
C7	0.0488 (17)	0.0427 (17)	0.0336 (17)	−0.0007 (14)	0.0050 (13)	−0.0005 (14)
C8	0.0461 (17)	0.0397 (17)	0.0336 (18)	0.0025 (13)	0.0080 (13)	0.0014 (14)
C9	0.0393 (15)	0.0412 (16)	0.0267 (16)	−0.0059 (13)	0.0007 (12)	0.0022 (13)
C10	0.0417 (16)	0.0453 (18)	0.052 (2)	−0.0023 (13)	−0.0060 (14)	0.0035 (15)
C11	0.060 (2)	0.0458 (19)	0.061 (2)	−0.0173 (16)	−0.0191 (18)	0.0191 (17)
C12	0.074 (2)	0.090 (3)	0.049 (2)	−0.041 (2)	−0.0053 (19)	0.025 (2)
C13	0.058 (2)	0.099 (3)	0.043 (2)	−0.024 (2)	0.0126 (16)	−0.001 (2)
C14	0.0478 (17)	0.062 (2)	0.0370 (18)	−0.0102 (15)	0.0069 (14)	−0.0009 (16)

### Geometric parameters (Å, °)

**Table:**

Cl1—C4	1.733 (3)	C5—C6	1.370 (4)
Cl2—C2	1.728 (3)	C5—H5	0.9300
Cl3—C11	1.726 (3)	C6—H6	0.9300
N1—C7	1.269 (3)	C7—H7	0.9300
N1—N2	1.375 (3)	C8—C9	1.487 (4)
N2—C8	1.347 (3)	C9—C14	1.374 (4)
N2—H2	0.896 (10)	C9—C10	1.383 (4)
O1—C8	1.222 (3)	C10—C11	1.368 (4)
C1—C2	1.382 (4)	C10—H10	0.9300
C1—C6	1.384 (4)	C11—C12	1.373 (5)
C1—C7	1.462 (4)	C12—C13	1.371 (5)
C2—C3	1.386 (4)	C12—H12	0.9300
C3—C4	1.365 (4)	C13—C14	1.377 (4)
C3—H3	0.9300	C13—H13	0.9300
C4—C5	1.369 (4)	C14—H14	0.9300
			
C7—N1—N2	116.1 (2)	N1—C7—H7	120.7
C8—N2—N1	117.7 (2)	C1—C7—H7	120.7
C8—N2—H2	122.6 (19)	O1—C8—N2	122.4 (3)
N1—N2—H2	119.5 (19)	O1—C8—C9	120.8 (3)
C2—C1—C6	117.1 (3)	N2—C8—C9	116.8 (3)
C2—C1—C7	122.4 (3)	C14—C9—C10	120.1 (3)
C6—C1—C7	120.5 (3)	C14—C9—C8	122.0 (3)
C1—C2—C3	122.1 (3)	C10—C9—C8	117.8 (3)
C1—C2—Cl2	120.4 (2)	C11—C10—C9	119.6 (3)
C3—C2—Cl2	117.5 (2)	C11—C10—H10	120.2
C4—C3—C2	118.3 (3)	C9—C10—H10	120.2
C4—C3—H3	120.8	C10—C11—C12	120.8 (3)
C2—C3—H3	120.8	C10—C11—Cl3	119.7 (3)
C3—C4—C5	121.5 (3)	C12—C11—Cl3	119.5 (3)
C3—C4—Cl1	119.2 (2)	C13—C12—C11	119.2 (3)
C5—C4—Cl1	119.2 (3)	C13—C12—H12	120.4
C4—C5—C6	119.1 (3)	C11—C12—H12	120.4
C4—C5—H5	120.4	C12—C13—C14	120.9 (3)
C6—C5—H5	120.4	C12—C13—H13	119.5
C5—C6—C1	121.9 (3)	C14—C13—H13	119.5
C5—C6—H6	119.0	C9—C14—C13	119.3 (3)
C1—C6—H6	119.0	C9—C14—H14	120.3
N1—C7—C1	118.5 (3)	C13—C14—H14	120.3

### Hydrogen-bond geometry (Å, °)

**Table:**

*D*—H···*A*	*D*—H	H···*A*	*D*···*A*	*D*—H···*A*
N2—H2···O1^i^	0.90 (1)	2.08 (2)	2.900 (3)	152 (3)
C7—H7···O1^i^	0.93	2.35	3.155 (3)	145

Symmetry codes: (i) *x*, −*y*+3/2, *z*+1/2.
